# Domestic waste emissions to European waters in the 2010s

**DOI:** 10.1038/s41597-020-0367-0

**Published:** 2020-01-23

**Authors:** Olga Vigiak, Bruna Grizzetti, Michela Zanni, Alberto Aloe, Chiara Dorati, Fayçal Bouraoui, Alberto Pistocchi

**Affiliations:** European Commission, Joint Research Centre (JRC), Ispra, Italy

**Keywords:** Pollution remediation, Water resources

## Abstract

Estimation of domestic waste emissions to waters is needed for pollution assessment and modelling. We assessed quantity and location of domestic waste emissions to European waters for the 2010s. Specifically, we considered discharges of domestic waste Population Equivalent (PE, the amount of waste that equals to 60 g per day of Biochemical Oxygen Demand), and mean annual loads (t/y) of total nitrogen, total phosphorus, and 5-days Biochemical Oxygen Demand. The spatial resolution and extent of the analysis corresponded to the CCM2 River and Catchment Database for Europe, for catchments of mean area of 6.4 km^2^. The assessment is based on available European databases that allowed pinpointing waste emissions to a high spatial and conceptual resolution. Content gaps, particularly concerning domestic waste from isolated dwellings, were filled through alternative sources of information, exploiting population density and national statistics data. The dataset is of interest for assessing waste emissions to and fate through European fresh and marine waters also beyond the three pollutants evaluated in this study.

## Abstract (summary)

**Table:** 

Measurement(s)	household waste material
Technology Type(s)	digital curation • computational modeling technique
Factor Type(s)	geographic location
Sample Characteristic - Environment	fresh water
Sample Characteristic - Location	Europe

Machine-accessible metadata file describing the reported data: 10.6084/m9.figshare.11559084

## Background & Summary

Estimation of domestic waste emissions to the European fresh and marine waters is needed for assessing current physico-chemical status of water bodies and providing inputs for pollutant transport and fate models. Estimation of domestic waste discharges requires assessment of spatial distribution of population and per capita pollutant emissions; treatment levels and pollutant removal efficiency; and location of waste discharge to waters^1^.

Continental and global studies generally rely on national statistics to assess domestic and industrial waste^1–3^. In Europe, regulatory efforts of the European Union^4,5^ prompted investments in waste treatment and as a result point source emissions to water bodies have declined^6^. Concurrently, reporting of waste emissions has been centralized through the European Environment Agency (EEA). Since 2010, the EEA collects and publishes data about domestic waste emissions reported by EU 28 Member States and other EEA member countries (Norway, Iceland, and Switzerland), providing detailed information about waste production and disposal locations. These data represent a detailed source of information of current emissions at high spatial resolution, however their spatial extent does not cover the entire European continent and some waste source gaps remain.

The aim of this study was to assess the quantity and location of domestic waste emissions of pollutants in European waters for the 2010s. Specifically, the pollutants considered in this study were domestic waste generation in terms of Population Equivalent (PE, i.e. the amount of waste that equals to 60 g per day of Biochemical Oxygen Demand), and emissions of total nitrogen (N), total phosphorus (P), and organic pollution as measured by 5-days Biochemical Oxygen Demand (BOD). Sources and pathways of these pollutants in the river basins were defined as follows. Most of the domestic waste produced by human settlements and common commercial activities are collected by sewerage systems and treated by waste water treatment plants (WWTPs) before being discharged to the surface water (connected and treated waste). The level of treatment can be primary (T1, mechanical removal), secondary (T2, biological removal), or tertiary (T3, advanced removal). Further, part of tertiary facilities may be equipped with removal systems for phosphorus (T3P). In some cases waste water is collected through sewer system but is not treated before being discharged (T0, connected not treated waste). Generally all households of an agglomeration are connected to the sewerage network. However, when this is not possible, Individual Appropriate Systems (IAS) can be in place. An IAS collects and treats domestic waste before releasing it to the environment or to a WWTP via truck transport. Small isolated houses (Scattered Dwellings, SD) are not connected to sewerage system but are generally equipped with septic tanks that remove part of the pollution load before waste infiltrates underground. In this assessment discharges from WWTPs (T1, T2, T3), and sewer pipes (T0) are considered direct emissions to surface water (point sources). Emissions from disconnected sources (IAS and SD) leach underground, where they could contribute locally to the pollution of groundwater, and reach surface water via subsurface pathways (diffuse sources).

**Fig. 1:**
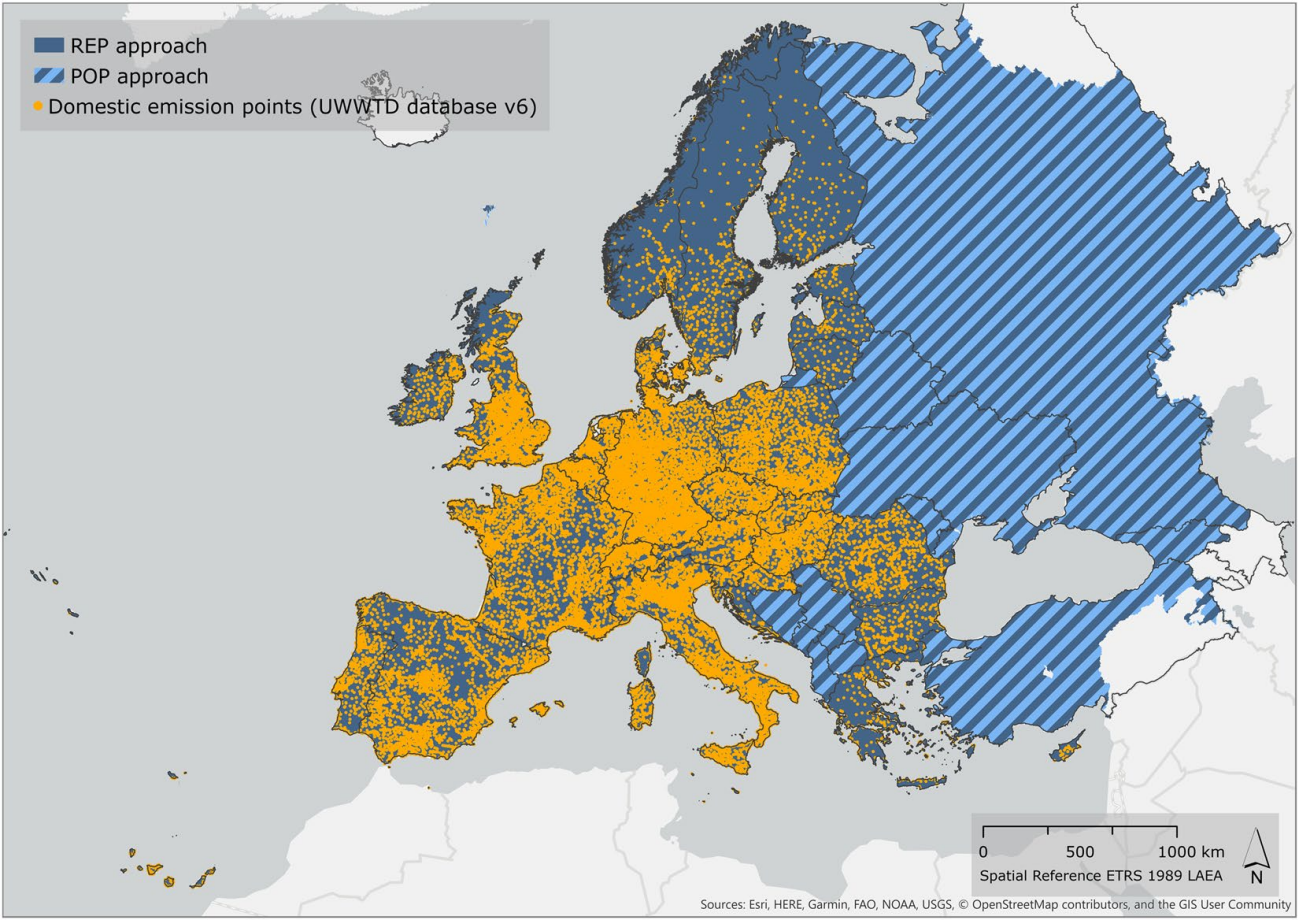
Domestic waste data coverage and extent of dataset. Orange dots indicate domestic emission points based on UWWTD database, covering EU28, Norway and Switzerland (REP approach). Blue background indicates the extent of the dataset and data coverage for population statistics (POP approach); stripes indicate regions for which solely POP approach data was available.

Homogeneous data on quantity and location of all sources of pollution outlined above are not available for the whole Europe. A method was developed to merge domestic waste data reported in databases (REP approach) with alternative sources of information based on population density and national statistics on sewerage connection and treatment level rates (POP approach), to fill in content and regional gaps (Fig. 1). The spatial resolution and extent of the analysis and dataset corresponded to the CCM2 River and Catchment Database for Europe^7–9^, which defines the European drainage network and divides the land into topologically connected catchments. Iceland was excluded from the analysis as it is not covered by CCM2. The reference period for the assessment was 2014–2015, although in some cases a longer time period was considered. The dataset is of interest to assess and model waste pollution in waters at the continental scale.

## Methods

The assessment of emissions of nitrogen, phosphorus and organic matter (BOD) in domestic waste involved several steps, namely assessment (i) of the spatial distribution of annual domestic waste (LOCATION); (ii) of the pollution loads (QUANTITY); and (iii) of the level of treatment (REDUCTION).

### Location

The Waterbase - Urban Waste Water Treatment Directive (UWWTD) database v6^10^, reporting data for 2014, was used as basis for domestic waste emissions in the REP approach region. The UWWTD database reports domestic waste emitted by agglomerations larger than 2000 Population Equivalent (PE) in the 28 European Union Member States plus Iceland, Norway, and Switzerland. The database reports waste loads generated in agglomerations, to which WWTP or IAS loads are transferred to, WWTP treatment levels, and location of WWTP discharge points. All loads are reported in terms of PE. Besides waste generated by resident population, PE loads comprise also commercial, industrial, or tourism waste that is produced in the agglomerations.

The UWWTD database is composed of several tables that portray the complex transfers between agglomerations, WWTPs, and discharge points. The waste load generated in agglomerations may be transferred to WWTPs, to IAS, or discharged without treatment. One agglomeration can be served by more than one WWTP and one WWTP may serve more agglomerations. Similarly, waste from IAS may be released in the environment or transferred in part or in total to one or more WWTPs by truck. Finally, a WWTP may have one or more discharge points.

In the database, some missing data and errors, for example in geographic coordinates, were detected. Further, inconsistencies between waste loads generated by agglomerations, transferred to WWTPs, treated, and ultimately transferred to discharge points were noted. Thus, the original database was amended, filling in missing information where possible and reducing inconsistencies by tracking records through the database structure, with the aim of preserving the generated waste load from agglomerations to treatment facilities and discharge points. Geographic coordinates were corrected where possible based on facility names, or location of linked items (agglomerations, WWTPs or discharge points). Rules applied to address the inconsistencies are detailed in Vigiak *et al*.^11^. Not all inconsistencies could be addressed; for example a discrepancy of 10% of waste between what was transferred to and received from any single WWTP was considered acceptable. While the revision process may have reduced database inconsistencies, it may also have inadvertently generated errors, as assumptions had to be made when addressing each inconsistency/error type. This was particularly true for Croatia, for which no information on the path of waste generated in agglomerations to IAS or WWTP was reported, and for which mean statistics of neighbour Slovenia were used instead. Thus, Croatia results should be considered approximations only.

The revision allowed to attribute PE generated in agglomerations (PE_GEN), to IAS or WWTPs discharge points. Waste load treated in IAS (PE_IAS) was equalled to the share of load transferred from agglomerations to IAS (TO_IAS) less the waste load transferred from agglomerations to WWTPs by truck (IAS_to_WWTP; Table 1). In total, about 627.5 million PE were generated in 2014 in the 30 countries comprised in the UWWTD database of the REP region (PE_GEN); of this waste, about 2.3% was not treated (PE_0), 1.8% was treated in IAS (PE_IAS), and 95.9% was connected and treated in WWTPs (PE_WWTP). Due to persistence of small inconsistencies at agglomeration or WWTP level, some small differences between PE generated and allocated to the three waste pathways remained, with the sum of allocations exceeding generated waste by 0.2% overall.

**Table 1:** Annual country waste load (in thousands of Population Equivalent, 10^3^ PE) resulting from the revision of UWWTD database adopted in this study. Waste load generated in agglomerations (PE_GEN) was in part not treated (PE_0), or destined to Individual Appropriate Systems (To_IAS). Load entering IAS could be transferred to WWTPs via truck (IAS_to_WWTP) or treated and discharged in the environment (PE_IAS). Load treated in WWTPS (PE_WWTP) comprises loads transferred directly from agglomerations or received from IAS.

Country	PE_GEN	To_IAS	IAS_to_WWTPS	PE_IAS	PE_0	PE_WWTP
	(10^3^ PE)	(10^3^ PE)	(10^3^ PE)	(10^3^ PE)	(10^3^ PE)	(10^3^ PE)
Austria	20,434.5	138.1	138.0	0.1	0.0	20,426.3
Belgium	9,243.8	0.0	0.0	0.0	23.1	9,212.5
Bulgaria	8,117.5	6.2	5.6	0.5	1,302.5	6,785.9
Croatia	5,026.2	259.5	0.0	259.5	366.3	4,398.5
Cyprus	995.0	16.2	1.2	15.0	240.5	739.5
Czech Republic	7,750.4	529.9	0.0	529.9	0.0	7,205.9
Denmark	11,612.5	0.0	0.0	0.0	0.0	11,577.9
Estonia	1,659.6	41.9	41.4	0.5	8.8	1,610.7
Finland	5,373.1	0.0	0.0	0.0	0.0	5,444.1
France	72,466.2	0.0	0.0	0.0	0.0	72,443.7
Germany	109,911.6	2,026.7	759.8	1,266.9	0.0	109,150.4
Greece	11,792.2	1,221.7	79.2	1,142.4	0.0	10,654.0
Hungary	11,880.5	1,527.2	0.0	1,527.2	0.0	10,329.6
Ireland	5,255.8	262.8	0.0	262.8	0.0	5,255.8
Italy	77,975.7	3,456.5	2.7	3,453.8	577.6	73,743.1
Latvia	1,572.9	85.7	0.0	85.7	0.0	1,499.7
Lithuania	2,665.0	128.7	0.0	128.7	0.0	2,539.2
Luxembourg	625.0	4.5	0.0	4.5	0.0	636.1
Malta	513.0	0.0	0.0	0.0	0.0	513.0
Netherlands	18,229.8	0.0	0.0	0.0	2.7	17,995.9
Norway	5,185.0	48.9	0.0	48.9	199.6	5,305.9
Poland	38,536.6	3,353.9	3,154.2	199.6	233.8	38,194.8
Portugal	12,105.6	0.0	0.0	0.0	6.1	12,100.0
Romania	23,423.7	152.8	89.5	63.3	10,549.6	12,823.5
Slovak Republic	4,656.3	766.4	1.5	764.9	14.3	3,867.2
Slovenia	1,472.0	92.1	0.0	92.1	126.4	1,254.7
Spain	64,484.0	937.8	0.0	937.8	883.8	62,675.1
Sweden	12,551.3	0.0	0.0	0.0	0.0	12,551.3
Switzerland	10,976.8	1.4	0.0	1.4	212.8	10,882.6
United Kingdom	70,973.7	371.2	78.8	292.5	0.0	70,820.9

Waste generated in agglomeration but not treated (PE_0) was considered to be discharged directly to the stream network at the agglomeration location, less a 10% abatement that occurs in the sewerage system^2^. As the UWWTD database reports no information about treatment and location of IAS, waste load treated in IAS (PE_IAS) was assumed to receive primary treatment and be discharged in the ground (diffuse source) at the agglomeration coordinates. Waste load treated in WWTPs (PE_WWTP) was reduced according to WWTP treatment level, and emitted in the stream network at the WWTP discharge points. When a WWTP had more than one discharge point, WWTP emissions were divided among discharge points assuming that larger portions of waste would be discharged to larger rivers/streams. The mean annual flow of the receiving reaches was used to define each discharge point receiving fraction.

In parallel and for the whole European continent, domestic waste was also assessed based on national statistics of domestic waste treatment coupled with the spatial distribution of population density (POP approach). This was the only method applicable in the region outside the 30 countries listed in Table 1. The percentage population connected to sewerage system and receiving waste water treatment level were derived from national statistics (Online-only Table 1). The main source of information was Eurostat^12^. Population shares were correspondingly defined as: (a) Collected in sewer (%, corresponding to Eurostat ‘Urban wastewater collecting system’); (b) IAS (%, ‘Independent wastewater treatment – total’); (c) 1ary treatment (%, ‘Urban and other waste water treatment plants - primary treatment’); (d) 2ary treatment (%, ‘Urban and other wastewater treatment plants - secondary treatment’); (e) 3ary treatment (%, ‘Urban and other wastewater treatment plants - tertiary treatment’). In a few instances, the distribution in 1ary, 2ary or 3ary treatment plants was unreported in Eurostat^12^, but was extracted instead from another Eurostat dataset^13^. From statistics (a) to (e), we derived three more population shares: (f) population whose waste is collected but not treated (Pop_0 = Collected in sewer − sum of 1ary, 2ary and 3ary treatments; = (a) − (c + d + e), %); (g) Disconnected population (DISC), i.e. population share whose waste is not collected in sewers (DISC = 100 − Collected in sewer, = 100 − (a), %); and (h) Scattered Dwellings (SD), i.e. small, sparsely distributed homesteads, equal to the share of disconnected population that is not treated with IAS (SD = DISC − IAS, (g − h) %).

Small inconsistencies in national statistics were identified. For example, IAS data were sometimes unreported or larger than DISC; Pop_0 did not always match Eurostat^12^ ‘Percentage of resident population not connected to urban and other waste water treatment plants’ statistics. The inconsistencies were addressed maintaining information about collected and treated shares, i.e. items from (a) to (e), while adjusting derived shares, from (f) to (h). These inconsistencies indicate a degree of conceptual uncertainty in defining population shares or in the interpretation assumed in this study, especially with regards to Pop_0 and DISC. When Eurostat^12^ data was not available other reporting sources were used (Online-only Table 1); however it was noted that different international sources indicated sometimes discordant figures^14^. Caution should be exerted especially for statistics reported for Albania, Moldova, and Russian Federation.

The 1 km^2^ raster grid of Global Human Settlement (GHS) population of 2015^15^ was used to define population density (inhabitants/km^2^). Population was allocated to waste water treatment shares according to its density, assuming that most densely populated areas would benefit of the best nationally available technology, and vice versa the least populated areas would not be connected to sewerage systems. Thus, four increasing population density thresholds per country were identified based on the national cumulative population density distribution and national treatment statistics as: Th_DISC_, below which density of population was assumed disconnected from sewerage; Th_0_ defining the density up to which population was assumed to be connected to sewers but whose waste was not treated; Th_1_ defining the density up to which population was assumed to be served by primary treatment; and Th_2_ defining the density up to which population was assumed to be served by secondary treatment; population densities above Th_2_ were assumed to be served by tertiary treatment. After applying the density thresholds, the number of inhabitants per treatment and per catchment was obtained by multiplying the catchment mean density (inhabitants/km^2^) for the relative share by the catchment area (km^2^). Through this procedure, population was spatially partitioned into:

i. Population that is not connected to sewer systems (Pop_DISC: GHS2015 population density < Th_DISC_). Pop_DISC was divided in the two fractions, Pop_IAS (i.e. whose waste is treated in IAS) and Pop_SD (i.e. waste generated in scattered dwellings, served by septic tanks). The ratio IAS/DISC between disconnected population whose waste is treated in IAS and all disconnected population was derived from national statistics and used to separate the two fractions: Pop_IAS = IAS/DISC * Pop_DISC; Pop_SD = (1 − IAS/DISC)*Pop_DISC (Online-only Table 1). Thus Pop_IAS and Pop_SD share the same spatial distribution.
ii. Population that is connected to sewer system but whose waste is not treated (Pop_0: Th_DISC_ > = density < Th_0_)
iii. Population that is connected to sewer system and whose waste is treated at primary (Pop_1: Th_0_ > = density < Th_1_), secondary (Pop_2: Th_1_ > = density < Th_2_), or tertiary level (Pop_3: density > = Th_2_).

A final check consisted of summing inhabitants per country and treatment level to see if proportions respected the official statistics. Deviations of allocated to official population shares were less than 0.35% in more than 90% of cases. The largest negative deviation was −3% for tertiary treatment in Turkey, and +3% of primary treatment in Georgia. Within EU28, the largest deviation was +1.2% population allocated to tertiary treatment in Luxemburg. These differences are due to errors in allocating a CCM2 catchment to a single country along country borders.

#### Merging of domestic waste data sources

The UWWTD database does not report waste from agglomerations below 2000 PE unless its waste is treated in WWTPs. Thus, part of the population that is disconnected from sewerage systems (part of Pop_DISC) or possibly served by sewerage but not treated in small agglomerations (part of Pop_0) remains unreported. To fill in this source gap it was necessary to estimate which quota of population might be unreported in the UWWTD database (called herein “residual population”, Pop_RES). This was done by relating domestic waste estimates from REP and POP approach. However, direct comparison was complicated by (i) differences in reported units, as the UWWTD database reports PE while the POP approach is based on inhabitants; and (ii) the highlighted uncertainties in reported shares of Pop_DISC and Pop_0 for the POP approach.

**Fig. 2:**
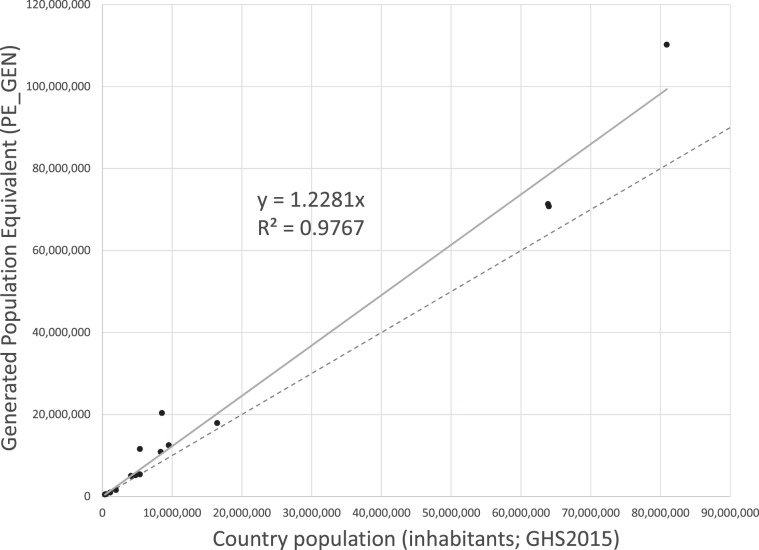
Relationship between generated population equivalent (PE_GEN) as reported in the UWWTD database and total population (inhabitants), for 15 countries that reported at least 97.5% of population as treated through IAS or WWTPs (Online-only Table 1).

The relationship between PE reported in the UWWTD database (PE_GEN) and inhabitants (total resident population PopTot, estimated from GHS2015^15^) needed be better understood to allow for a meaningful merging of the two approaches. Theoretically, missing population in the UWWTD database would be negligible in countries where shares of scattered dwellings (disconnected but not treated in IAS) or connected but not treated (Pop_0) population is nil or very low. Of the 30 countries analysed in the UWWTD database, 15 reported at least 97.5% of population as treated through IAS or WWTPs (Online-only Table 1). The country ratio between PE_GEN and PopTot (inhabitants) for these 15 countries ranged from 0.8 to 2.4 (median 1.18). Despite this variability and the small sample size, a significant linear regression between PE_GEN and PopTot could be identified: PE_GEN = 1.23 inhabitant (R^2^ = 0.98; sample size = 15; Fig. 2). The rate PE/inhabitant of 1.23 was thus adopted to transform PE into resident population and vice versa. We refer to inhabitants derived from PE as Population Resident Equivalents (PRE, inhabitants), where 1 PRE = 1 PE /1.23. The interpretation of this rate is that on average across Europe the contribution of commercial, industrial and tourism emissions to domestic waste on top of resident population can be considered around 23%. This figure is higher than a global average of 15%^2^ but seems reasonable for industrialized countries, and especially for urban areas.

**Fig. 3:**
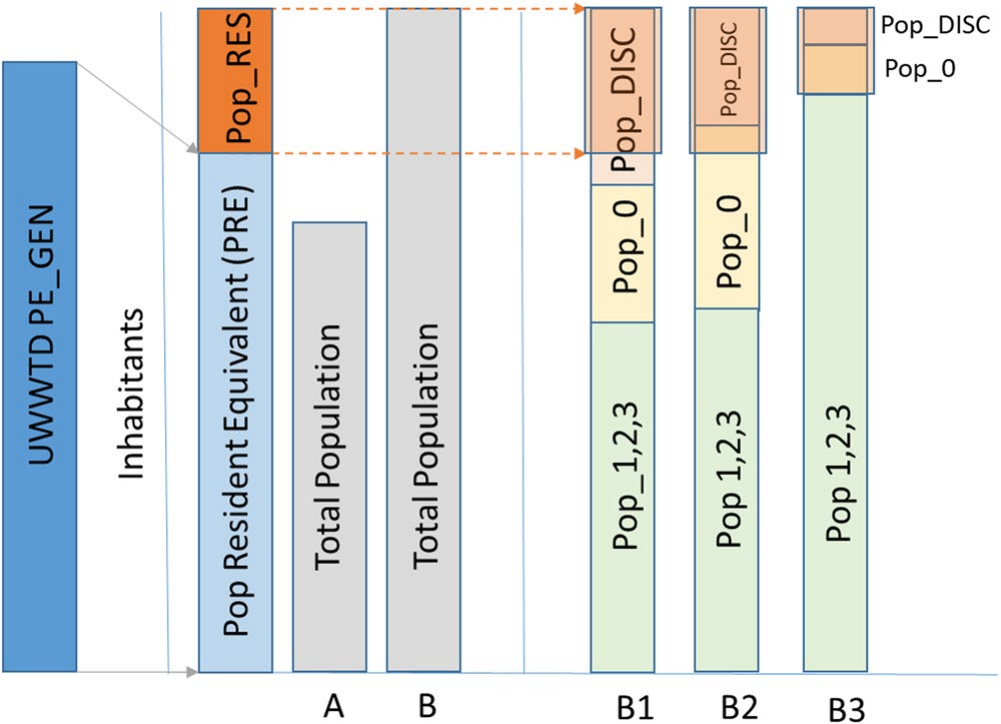
Conceptual scheme of the procedure for assessing population unreported in the UWWTD database (by country) by comparing reported PEs with resident population in the GHS 2015 population dataset. PE_GEN: total generated Population Equivalents per country. PRE: the equivalent amount in Population Resident Equivalent (PRE = PE/1.23; inhabitants). A, B, B1, B2, and B3 represents different cases per European countries (explanation in the text).

The PE/inhabitant rate was used to estimate the quota of country population that was not accounted for in the UWWTD database (Pop_RES). Figure 3 shows a conceptual scheme of the procedure. First, country total PE_GEN reported in the UWWTD database (blue bar) was transformed into population resident equivalents (PRE), which were compared to total population (PopTot). The difference between total population (PopTot) and the estimated inhabitants reported in the UWWTD database (PRE) was defined Pop_RES. If the total population was lower than estimated PRE, Pop_RES was nil (case A in Fig. 3). Otherwise (cases B in Fig. 3), Pop_RES was taken (and spatially distributed) as part of population disconnected (Pop_DISC) or connected but not treated (Pop_0). In first instance, if disconnected population was larger than the estimated Pop_RES (case B1 in Fig. 3), then Pop_RES was defined and distributed as the Pop_RES/Pop_DISC fraction of the disconnected population; all this fraction was considered belonging to scattered dwellings (Pop_SD). When Pop_RES was larger than Pop_DISC (case B2 in Fig. 3), then after allocating Pop_SD equal to Pop_DISC, the remaining portion of Pop_RES was taken and distributed as a fraction of connected not treated population (Pop_RES_0). Finally, there could be cases where Pop_RES was larger than the sum of Pop_DISC and Pop_0 (case B3 in Fig. 3). In these cases, all Pop_DISC was considered Pop_SD, all Pop_0 was considered Pop_RES_0, but there was no further attempt to fill the remaining estimated population gap, and the final Pop_RES allocated to the country was lower than the population gap initially estimated.

In some countries (AT, CH, DE, DK, EE, ES, IT, MT, and SE; country codes are indicated in Online-only Table 1), PRE exceeded population thus Pop_RES was nil. In other cases (BE, CY, CZ, LT, LV, PL, SI, and SK), population exceeded PE_GEN; in these countries Pop_RES amounted to 16–42% of population, and was a considerable source of domestic waste in addition to what reported in the UWWTD database. Finally, in the remaining countries (BG, FI, FR, GB, GR, HR, HU, IE, LU, NL, NO, PT, RO) total PE_GEN were larger than population, but the corresponding PRE were lower than population. In these cases, the median Pop_RES was 3% of population, but was larger in FR (9% of population), NO (11%), and FI (17%). In total, Pop_RES amounted to about 25 million inhabitants.

In the POP approach, shares of connected population (Pop_0 to Pop_3) were transformed into PE loads using the PRE equivalence definition, i.e. adding a 23% component due to commercial, industrial and tourism to that of resident population. However, a 10% reduction was applied to these PE loads to account for losses occurring in sewerage system^2^. Pop_IAS were also transformed into PRE, adding a 23% to align them to PE_IAS definition. Instead, domestic load from scattered dwellings (Pop_SD) was considered as produced solely by resident inhabitants (1 PE/inhabitant in this case).

In synthesis, the two datasets (REP and POP) were merged as follows (Fig. 1):

- Treated loads: In regions covered by the UWWTD database, treated load was estimated with the UWWTD database and attributed to discharge point locations (REP approach). For countries not covered by the UWWTD database, treated waste was estimated with Pop_1, Pop_2 and Pop_3 assessed with POP approach, and emissions were distributed according to catchment population density;
- Disconnected and connected not treated domestic loads: In regions covered by the UWWTD database, PE_IAS and PE_0 reported in the UWWTD database were attributed at agglomeration coordinates. Additionally, unreported population pertaining to small agglomerations (Pop_RES) was distributed according to POP approach (Pop_SD and Pop_RES_0). For countries not covered by the UWWTD database, the analogues from POP approach (Pop_SD, Pop_IAS, and Pop_0) were used.

### Domestic pollutants loads (QUANTITY)

All waste generated by these sources was considered domestic, although urban waste reported in the UWWTD database includes waste from commercial, industrial, and tourism activities. After allocating domestic waste load (PE) spatially across Europe, the associated emissions of nitrogen, phosphorus, and BOD loads were estimated assuming them to be dependent on human diet^1,16,17^.

Emissions of nitrogen and phosphorus from human excreta were estimated based on protein consume^16^. Consume was considered equal to intake less a 20% of retail losses for vegetable proteins and 11% for animal proteins, and a further 3% of losses through sweat/hair/blood^2^. Therefore, N and P emissions (E_N_ and E_P_; g/day/PE) were calculated according to Jönsson and Vinnerås^16^ as:

1 $${{\rm{E}}}_{{\rm{N}}}=(1-0.03)\ast {{\rm{\alpha }}}_{{\rm{N}}}\ast ({\rm{VEGPRT}}\ast (1-0.2)+{\rm{ANIMPRT}}\ast (1-0.11))$$

2 $${{\rm{E}}}_{{\rm{P}}}=(1-0.03)\ast {{\rm{\alpha }}}_{{\rm{P}}}\ast (2\ast {\rm{VEGPRT}}\ast (1-0.2)+{\rm{ANIMPRT}}(1-0.11))$$

where VEGPRT is the vegetable protein intake, ANIMPRT is the animal protein intake (g/day/PE); α_Ν_ is content of nitrogen in proteins (0.11) and α_P_ is the content of phosphorus in proteins (0.011)^16^. Protein intake was derived from FAO statistics of 2009–2011^18^. Emissions of BOD (E_BOD_) were assumed equal to 60 g/day/PE as per UWWTD database definition, although BOD emissions in Europe may vary from 40 to 70 g/day/PE depending on diet^3^. Finally, daily emissions were transformed into annual loads (Load_in,X_, t/y):

3 $${{\rm{Load}}}_{{\rm{in,X}}}={{\rm{E}}}_{{\rm{X}}}\ast 365.25/1000000$$

where X indicates the constituent (nitrogen, phosphorus or BOD). An additional source of phosphorus emissions in domestic waste due to use of detergents was estimated with Bouraoui *et al*.^1^ (Online-only Table 1).

### Treatment pollution removal (REDUCTION)

Annual load emissions of domestic waste were computed as:

4 $${{\rm{Load}}}_{{\rm{out}},{\rm{T}},{\rm{X}}}={{\rm{Load}}}_{{\rm{in}}{\rm{T,X}}}(1-{{\rm{eff}}}_{{\rm{T,X}}})$$

where Load_out,T,X_ is the annual pollutant emission load of constituent X (t/y of nitrogen, phosphorus, or BOD) at treatment level T; Load_in,T,X_ is the annual load undergoing treatment, and eff_T,X_ is the removal efficiency. Removal efficiencies per treatment level were adopted from literature^3,19–21^ (Table 2). BOD efficiencies were set after calibration of BOD fluxes in Europe^22^ within the range of literature values.

**Table 2:** Treatment removal efficiencies adopted in this study per treatment level T and constituent X (N = nitrogen; P = phosphorus; BOD = Biochemical Oxygen Demand).

Treatment level	Applied to	N	P	BOD
Septic Tank	Pop_SD	0.25	0.30	0.40
T1 - Primary	PE_IAS; PE_1	0.25	0.30	0.50
T2 - Secondary	PE_2	0.55	0.60	0.94
T3 - Tertiary	PE_3	0.80	0.60	0.96
T3P - Tertiary with phosphorus removal	PE_3P	0.80	0.90	0.96

WWTP treatment types are reported in the UWWTD database^10^, however only nutrient removal technologies were considered to assign tertiary treatment level. WWTP treatment levels for nutrient were thus assigned as follows: tertiary when nitrogen or phosphorus removal was indicated; secondary when secondary treatment was specified, primary in all other cases. Noteworthy, in this way primary level was assigned to 1424 WWTPs which had no treatment reported in the UWWTD database, and to further 394 WWTP ambiguous cases.

Phosphorus removal technology improves tertiary treatment P efficiencies sensibly (T3P; Table 2). In the UWWTD database adoption of phosphorus removal is specified. In the POP approach, national statistics report this information partially^12^. However, the fraction of tertiary WWTPs that include phosphorus removal in the UWWTD database was generally higher than Eurostat^12^ data, possibly because the UWWTD database is more recent. Thus the rate of phosphorus removal adoption in tertiary treatment (T3P/T3) as estimated from UWWTD database was adopted to define the fraction of Pop_3 treated with phosphorous removal (Pop_3P = Pop_3*T3P/T3). In countries not covered by UWWTD database, for which no data was available, all tertiary treatment was considered without phosphorus removal technology (T3 only; Pop_3P = 0).

## Data Records

Waste emissions to waters comprised point and diffuse sources. Locations of individual point sources however are subject to some uncertainty, thus we elected to aggregate emissions at the CCM2^9^ catchment scale, which we consider appropriate for modelling pollution transport in freshwaters at continental scale. Further, emissions were aggregated at administrative units, which comprised NUTS Level 3 European administrative units^23^, united to ESRI administrative units^24^ for countries not covered by Eurostat^23^.

The dataset produced in this study^25^ consists of the original CCM2 catchment polygon layer^7–9^ (spatial projection ETRS LAEA 1989) whose table reports emissions to waters according to the items listed in Online-only Table 2. All sources of the same type (table items) were aggregated per catchment. The list of items reported in Online-only Table 2 reflects the approach and interpretation given in this study. To allow for a more flexible use of the dataset, for example in case users would select only some waste sources, or apply the dataset to other waste related studies, we report domestic waste in terms of PE load emitted through different pathways and derived constituents (nitrogen, phosphorus, and BOD) as estimated in our study. For the administrative units, we provide the same table but with reference to administrative regional code at NUTS Level 3^23^.

## Technical Validation

### Comparison of domestic waste population shares

Continental and global assessments of domestic waste are usually based on population and national statistics as availability of more detailed data is rare. Thus, it is insightful to compare country domestic PE loads for the region where both REP and POP approaches could be applied.

**Fig. 4:**
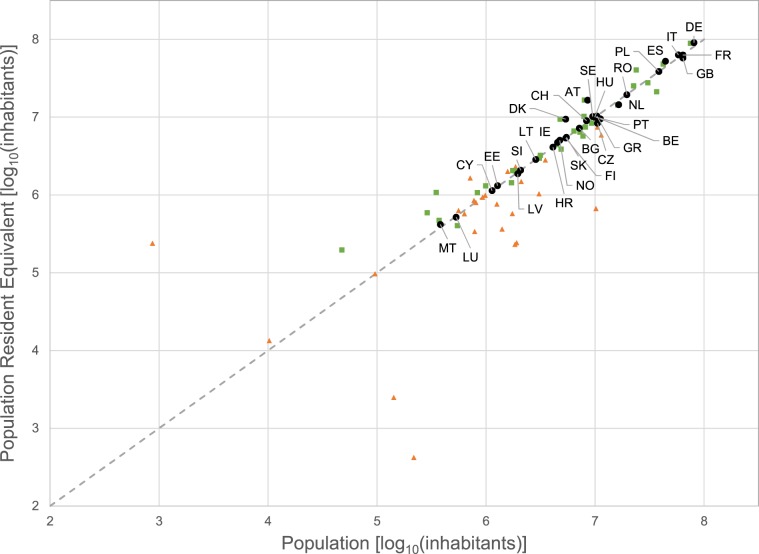
Comparison of country Population Resident Equivalent (PRE; in log_10_[inhabitants]) estimated in the REP approach against population (log_10_[inhabitants]) as derived from GHS2015. Dashed line indicates 1:1 relationship. Black dots = total population; orange triangles = disconnected population; green squares = tertiary treatment level.

Country total Population Resident Equivalent in the two approaches showed very good agreement, with a Pearson’s correlation coefficient ρ of 0.99 (Fig. 4; coefficient of determination R2 = 0.98; percent bias PBIAS = 4.6%; Nash-Sutcliffe Efficiency NSE = 0.98). This was achieved thanks to the introduction of ‘residual population’ to account for small dwellings that may be unreported in the UWWTD database. Yet, in some countries, notably AT and DK, which reported large PE loads, PRE in the REP approach remains higher than residential population and above the 1:1 line. Conversely, others countries, like BE and CZ, reporting lower than expected PEs but small shares of disconnected or untreated population, lay below the 1:1 line.

Larger differences amongst the two approaches emerge however when looking at shares of population by treatment level. The correlation ρ between PRE in disconnected population was 0.78 (Fig. 4, orange triangles; R2 = 0.61; PBIAS = −4.6%; NSE = 0.46). This attests inconsistencies in data sources used in the two approaches. For example, the GB share of disconnected population in REP approach is much higher than in POP, because the UWWTD database reports that in GB almost 300,000 PE are treated via IAS (part of disconnected population, Table 1) whereas Eurostat^9^ reports all population as connected and treated (Online-only Table 1). At the same time REP data for CH, EE, RO reports lower shares of disconnected population but higher shares of connected not treated population than POP data. The highest inconsistency between the two approaches was observed for ‘Connected not treated’ population share (PE_0/Pop_0). For this share, the largest deviation was the case of IT; in the UWWTD database, IT reports 0.7% of connected not treated PE (Table 1), but Eurostat^9^ reports about 30% of population as connected to sewerage but not treated (Online-only Table 1).

Correlation increased for primary treatment share, albeit differences for some countries were large (Pearson’s correlation coefficient ρ = 0.79; R2 = 0.62; PBIAS = +10.6%; NSE = −0.41). In this case, part of the discrepancies between the two approaches may have been generated by assuming primary treatment for WWTPs whose treatment type was not clearly declared in the UWWTD database. The assumption affected the majority (>75%) of WWTPs classified as primary treatment in BE, BG, CZ, ES, FI, HR, IE, LU, LV, RO, SI and SK. Conversely, it did not affect REP primary treatment for DE, GB or DK, which instead report larger shares of primary treatment through the UWWTD database than through national statistics (Table 1 and Online-only Table 1). The agreement between shares of population increases substantially in secondary (ρ  = 0.98; R2 = 0.95; PBIAS = 3.5%; NSE = 0.69) and tertiary treatment (ρ = 0.96; green squares in Fig. 4; R2 = 0.93; PBIAS = 8.8%; NSE = 0.89). This is reassuring because shares of domestic loads treated at the secondary or higher level represent the large majority of population (89% of PE_GEN).

Figure 4 highlights discrepancies in national reporting of domestic waste through different channels^14^. It is possible that interpretation of Eurostat statistics by reporting countries was different from the one assumed in this study (Online-only Table 1). Differences in reporting periods and variability in the PE/Population rate further complicate comparisons.

**Fig. 5:**
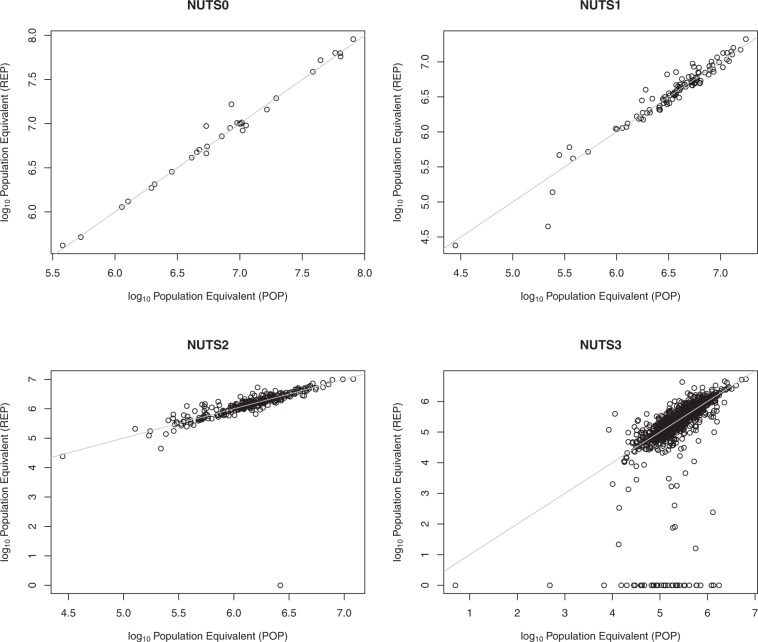
Amount of domestic waste [log10(PE)] attributed with the REP approach (based on UWWTD database point information, Y axis) or the POP approach (based on population density and national statistics, X axis) to spatial features of different administrative scales: NUTS Level 0 (Country), NUTS level 1; NUTS Level 2 and NUTS level 3.

Finally, we would like to remark spatial distribution implications in using waste emissions from point data (REP approach) or from combining population density and national statistics (POP approach). Figure 5 compares PE loads (in logarithmic scale) estimated by the two approaches and aggregated at decreasing spatial administrative scale, from country level (NUTS Level 1), to NUTS Level 2, and NUTS Level 3. At national scale the two approaches are consistent and close to 1:1 line. However, allocation of domestic waste diverges under the two approaches as the spatial scale gets finer. Pearson’s correlation coefficient ρ decreased from 0.99 at country scale (NUTS Level 0), to 0.96 at NUTS Level 1, 0.93 at NUTS level 2, to 0.83 at NUTS Level 3 (and to 0.34 at CCM2 catchment scale). A limit case at NUTS Level 2 scale exemplifies the cause of the divergence: UKI4, part of greater London, where 2.6 million people live, scores 0 PEs in the REP approach because no WWTP discharge point falls in this area, whereas under the POP approach 3.2 million PEs are attributed to this region.

Both approaches suffer limits and uncertainties, raising on one side from the assumptions adopted in the study, and on the other side from errors and inconsistencies that were detected in data sources, thus discrepancies should not to be regarded as errors in either approach. Yet, the UWWTD database represents an important step forward in tracking domestic waste generation and fate, and reporting quality of the original UWWTD database has improved in time. At the same time, the introduction of “residual population” shares allowed to align the two approaches and to fill in a domestic source gap (small dwellings) in the UWWTD database.

### Comparison of emission loads and removal efficiencies with UWWTD database data

For a minority of WWTPs and on voluntary basis, the UWWTD database reports incoming and exiting loads of nitrogen, phosphorus and BOD. When the reported loads were consistent with incoming PE load and WWTP treatment level was unambiguously declared, this information was used to (i) test the estimation of pollutant loads from domestic waste adopted in this study, and (ii) compare reported treatment efficiencies with those assumed in this study (Table 2). Notably however, declared WWTPs emissions were not retained in the final dataset^25^ to avoid methodological inconsistencies, which could rise for example when using the dataset for scenarios analysis.

**Fig. 6:**
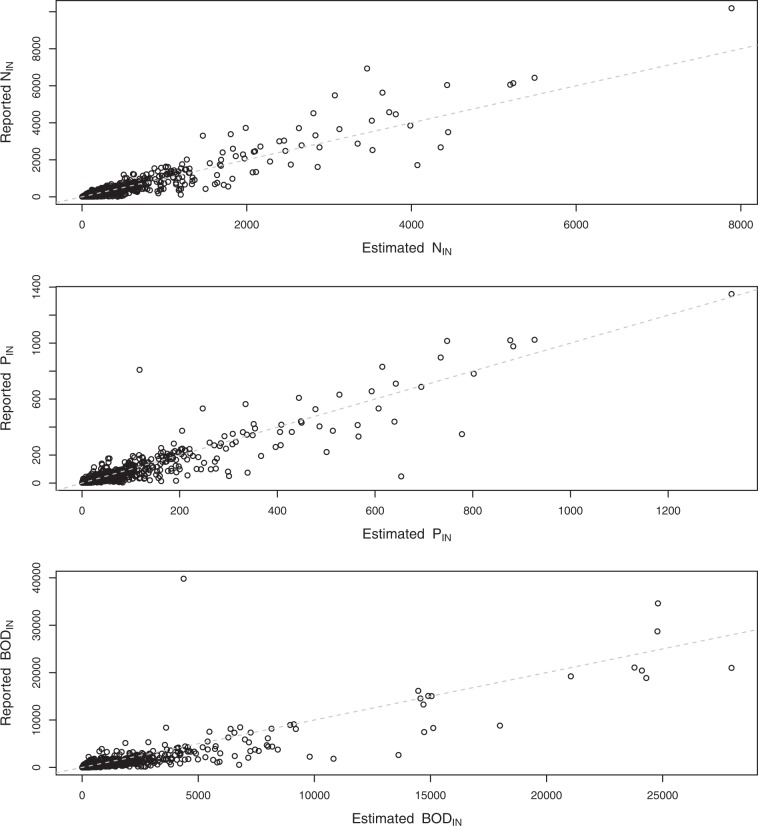
Comparison of pollutant incoming loads (nitrogen N, phosphorus P and Biochemical Oxygen Demand BOD; in t/y) reported in the UWWTD database (y axis) and the corresponding loads estimated based on human diet (x axis). The dashed grey line indicates 1:1 relationship. Sample sizes are reported in Table 3.

In Fig. 6, declared incoming loads as reported in UWWTD database are compared with incoming loads as estimated from human diet in this study. The 1:1 line indicates that the estimated incoming loads are in good agreement with those declared; linear regression coefficients were 1.08 for nitrogen (AdjR^2^ = 0.90; goodness of fit measures for the 1:1 relationship were R2 = 0.89; PBIAS = -4.2%; NSE = 0.88), 0.94 for phosphorus (adjR^2^ = 0.87; R2 = 0.85; PBIAS = 3.3%; NSE = 0.85) and 0.83 for BOD (AdjR^2^ = 0.74; R2 = 0.72; PBIAS = 29.9%; NSE = 0.68).

Reported treatment efficiencies varied considerably, with interquartile ranges being larger than 0.2 (Table 3). Mean efficiencies from reported data however compare very well with the ones assumed in this study (Table 2). Assumed treatment efficiency for BOD at secondary level and for phosphorus removal were slightly higher than reported means, but were still within the interquartile ranges. In any case, Table 3 indicates that removal efficiencies are a substantial source of uncertainty in the estimation of domestic waste emissions.

**Table 3:** Removal efficiencies in WWTPs reported in the Waterbase-UWWTD database^10^. UWWTD database data was retained when the reported loads were consistent with incoming PE load and treatment level was unambiguously declared thus WWTP sample size (#) is smaller than that reported in the original UWWTD database. IQR = Interquartile range, NA = Not applicable. N = nitrogen, P = phosphorus, BOD = Biochemical Oxygen Demand.

Treatment level		N	P	BOD
T1 – Primary	# WWTPs	11	5	33
	Mean Efficiency	0.36	0.36	0.50
	IQR efficiency	0.19–0.51	0.10–0.50	0.25–0.75
T2 - Secondary	# WWTPs	1214	570	841
	Mean Efficiency	0.50	0.59	0.90
	IQR efficiency	0.39–0.71	0.50–0.71	0.68–0.95
T3 - Tertiary	# WWTPs	5774	596	1859
	Mean Efficiency	0.77	0.61	0.97
	IQR efficiency	0.70–0.90	0.50–0.75	0.94–0.99
T3P - Tertiary + phosphorus removal	# WWTPs	NA	3143	NA
	Mean Efficiency		0.82	
	IQR efficiency		0.75–0.92	

### Comparison of WWTP emissions from different sources

The European Pollutant Release and Transfer Register database (E-PRTR)^26^ reports emissions from large WWTPs (i.e. those with incoming loads above 100,000 PE or whose emissions are higher than given thresholds). The presence of WWTPs in the E-PRTR database provides another source of information about pollutant emissions of domestic waste, albeit limited to a sample of very large facilities. An independent study^27^ analysed WWTP-related information reported in the E-PRTR and estimated median emission factors for nitrogen (N), phosphorus (P) and Total Organic Carbon (TOC) per PE and per treatment level, which can be compared to this study estimations (Table 4).

**Table 4:** Comparison of emission factors of nitrogen (N), phosphorus (P) and Total Organic Carbon (TOC) per PE estimated by (i) an independent study^27^ based on E-PRTR data^26^; (ii) in this study; and (iii) in the UWWTD database^10^ subset of data.

		Based on E-PRTR database^27^		Estimated in this study	UWWTD database^10^	
		Median emission factor	Sample size	(Section 2.2)	Emission factor	Sample size
		kg/PE/y	#	kg/PE/y	kg/PE/y	#
N	T1	2.41	19	2.61	1.73	11
	T2	1.85	432	1.56	1.38	1214
	T3	0.83	812	0.70	0.78	5774
P	T1	0.2	21	0.53	0.18	5
	T2	0.17	416	0.31	0.15	570
	T3	0.08	714	0.30	0.16	596
	T3P			0.07	0.06	3143
TOC	T1	5.75	10	6.52 (5.33–8.40)*	3.49 (2.85–4.49)*	33
	T2	1.16	397	0.78 (0.64–1.01)*	0.98 (0.80–1.27)*	841
	T3	0.88	805	0.52 (0.43–0.67)*	0.49 (0.40–0.63)*	1859

*TOC was estimated from BOD assuming a ratio BOD/TOC = 1.68 +/− 0.375 after Dubber and Gray^28^. Values in brackets report TOC emission ranges when adopting this error.

Nitrogen emissions estimated in this study are slightly higher than medians reported in van Duijnhoven and van den Roovart^27^ or emissions declared in the UWWTD database, probably because of the low efficiency assumed for primary treatment (Table 2). Conversely, phosphorus emissions for primary and secondary treatment estimated in this study were higher than what reported in the E-PRTR or UWWTD database. van den Roovart *et al*.^27^ did not separate tertiary treatment with or without P removal. However, most of tertiary WWTPs include phosphorus removal technology (80% of tertiary facilities, treating 90% of incoming waste load treated at tertiary level according to UWWTD database). van den Roovart *et al*. study^27^ emission factors concur with this study estimates and UWWTD reported emissions for phosphorus removal level T3P.

To transform BOD into TOC, this study assumed the molecular ratio of 1.68 +/− 0.375^28^. For primary treatment, TOC estimations of this study concur with median emissions from E-PRTR, but are higher than estimates from the UWWTD database data. Conversely, at secondary or tertiary treatment this study estimates concur with UWWTD database but are lower than van den Roovart *et al*.^27^ estimates. The comparison confirms the validity of assumptions taken in this study to assess emission loads and abatement, but highlights as well the uncertainty in the estimation of emissions.

## Usage Notes

Emissions to waters (items in Online-only Table 2) were selected to allow for flexible usage. PE loads of domestic waste can be used for assessing other forms of waste pollution, for example emergent pollutants. PE for the REP approach conforms to UWWTD database definition; PE in POP approach areas were derived multiplying population by the 1.23 factor assumed for Europe. As they are not part of official reporting, shares of domestic waste for “residual population” were kept separate to allow users to discard them should they prefer that. Users can decide whether to accept this study methods to assess nitrogen, phosphorus or BOD emissions, or to change some assumptions, in e.g. treatment efficiencies, pathways, etc. For example Morée *et al*.^2^ applied a 20% of losses on incoming nitrogen loads in scattered dwellings for volatilization; this loss was not applied in the present dataset but can be easily enforced should the user want to.

The database can be downloaded as an ESRI file geodatabase that includes catchment spatial information, or as stand-alone tables (csv format). In addition to the database, a map viewer allows browsing the dataset according to the four constituents (PE, nitrogen, phosphorus, or BOD, in annual values per km^2^) and three types of nested hierarchies: administrative units^23,24^, hydrological units^9^, and official Water Framework Directive River Basins Districts^29^. The web-viewer is scale dependent, so units activate or deactivate by zooming in or out of an area, and can be used to explore amount and shares of PE, and loads of nitrogen, phosphorus and BOD emissions. The default viewer displays PE at administrative level. Three main buttons on the right side of the viewer allows to activate the legend, change layers to be displayed (top layer selected covers the others), and access an information page. By clicking on any unit, a pop-up window opens to show dataset content.

We discourage spatial disaggregation of the reported loads to scale larger than the CCM2 catchments to limit the impact of potential location errors on tracking emissions. Population density used in the POP approach has an original scale of 1 km^2^. For REP region, should users wish to revert to point discharges, then the original UWWTD database^10^ could be used instead of this dataset.

## Data Availability

UWWTD database changes were developed through several R scripts whereas checks and corrections of spatial coordinates were conducted using ArcGIS interface. Final assembly of GIS dataset was conducted using SQL scripts. We regret it is not possible to package all the codes and procedure into sharable documentation.

## Online-only Tables

**Online-only Table 1:** National shares (%) of population connected to sewers, to Independent Appropriate Systems (IAS; when the data was not available, i.e. IAS/DISC = NA, IAS share was assumed nil) and to wastewater treatment levels (primary T1, secondary T2 or tertiary T3) adopted in the POP approach. The reference year was 2015, or closest possible (indicated in Data Year). Percentages of scattered dwellings (SD) or collected but not treated (T0) shares are derived from the others.

Country	CODE	Data year	IAS/DISC	Collected in sewer	SD	IAS	T0	T1	T2	T3	Phosphorus from detergents^+^	Data sources
			(%)	(%)	(%)	(%)	(%)	(%)	(%)	(%)	(kg P/person/day)	
Albania^^^	AL	2015	NA	19.0	81.0	0.0	0.0	11.0	7.0	1.0	0.2350	^12^
Austria°	AT	2014	100	95.0	0.0	5.0	0	0.0	1.2	93.8	0.0948	^12^
Belarus^(a)^	BY	2015	NA	91.1	8.9	0	0.7	25.7	64.7	0.0	0.0897	^30, 31^
Belgium	BE	2013	100	91.4	0.0	8.6	7.2	0.0	10.8	73.4	0.0821	^12^
Bosnia Herzegovina	BA	2013	NA	35.2	64.8	0	33.3	0.1	1.2	0.6	0.4510	^12^
Bulgaria	BG	2015	100	75.5	0.0	24.5	13.1	1.7	16.9	43.8	0.1162	^12^
Croatia°	HR	2015	100	54.6	0.0	45.4	1.7	16.0	35.9	1.0	0.5271	^12^
Cyprus°	CY	2005	100	29.8	0.0	70.2	0.0	0.0	4.7	25.1	0.5496	^12, 13^
Czech Republic	CZ	2015	16	85.2	14.4	2.4	4.2	0.2	6.9	73.9	0.2843	^12^
Denmark°	Dk	2014	100	91.0	0.0	9.0	0.0	0.0	2.0	89.0	0.1706	^12^
Estonia	EE	2014	29	83.0	12.0	5.0	0.0	0.0	5.0	78.0	0.1706	^12^
Finland°	FI	2013	100	83.0	0.0	17.0	0.0	0.0	0.0	83.0	0.1327	^12^
France°	FR	2014	100	82.1	0.0	18.0	1.6	0.1	14.3	66.1	0.3664	^12^
Georgia	GE	2015	42	44.2	32.4	23.4	12.1	28.6	3.3	0.2	0.1162	^32^
Germany°	DE	2013	84	96.2	0.6	3.2	0.8	0.0	2.5	92.9	0.1074	^12^
Greece	GR	2014	0	92.9	7.1	0.0	0.0	0.0	3.6	89.3	0.3032	^12^
Hungary	HU	2015	NA	78.8	21.2	0.0	1.9	0.1	12.2	64.6	0.2274	^12^
Ireland	IE	2014	100	69.0	0.0	31.0	4.0	0.0	47.0	18.0	0.0569	^12^
Italy	IT	2015	NA	94.0^$^	6.0	0.0	31.5	2.9	18.7	40.9	0.0632	^12^
Kosovo*	XK	2015	0	54.3	45.7	0.0	53.7	0.0	0.6	0.0	0.2350	^12^
Latvia°	LV	2013	100	71.1	0.0	28.9	0.2	3.7	50.0	17.2	0.1958	^12^
Lithuania	LT	2015	NA	72.5	27.5	0.0	0.1	0.1	6.9	65.4	0.1895	^12^
Luxembourg°	LU	2015	100	98.2	0.0	1.8	0.0	1.8	25.2	71.2	0.0948	^12^
Macedonia, FYR^(b)^	MK	2012	NA	60.0	40.0	0.0	47.0	6.5	6.5	0.0	0.2350	^33^
Malta°	MT	2014	0	98.6	1.4	0.0	0.0	6.4	92.2	0.0	0.3791	^12^
Moldova^(b,^ ^^)^	MD	2013	NA	38.0	62.0	0.0	14.0	12.0	12.0	0.0	0.0459	^34^
Montenegro^(b)^	ME	2012	NA	43.0	57.0	0.0	25.0	9.0	9.0	0.0	0.2350	^35^
Netherlands°	NL	2015	100	99.4	0.0	0.6	0.0	0.0	1.0	98.4	0.0821	^12^
Norway°	NO	2015	100	86.2	0.0	13.8	2.4	18.1	1.6	64.1	0.1137	^12^
Poland	PL	2015	NA	72.6	27.4	0.0	0.0	0.0	13.7	58.9	0.5244	^12^
Portugal	PT	2009	27	81.3	13.7	5.0^#^	21.9	3.6	39.4	16.4	0.4043	^12^
Romania	RO	2015	4	47.8	50.1	2.1	1.9	6.3	14.7	24.9	0.0897	^12^
Russian Fed. ^	RU	2000	NA	75.0	25.0	0.0	18.0	2.0	54.0	1.0	0.0897	^36^
Serbia	RS	2015	NA	58.7	41.3	0.0	46.8	1.3	8.7	1.9	0.2350	^12^
Slovak Republic	SK	2015	NA	65.2	34.8	0.0	0.7	0.1	33.0	31.4	0.1579	^12, 13^
Slovenia	SI	2015	94	62.6	2.2	35.2	5.0	0.0	30.5	27.1	0.1074	^12^
Spain	ES	2014	54	97.2	1.3	1.5	2.6	1.7	23.9	69.0	0.3854	^12^
Sweden°	SE	2014	100	87.0	0.0	13.0	0.0	0.0	4.0	83.0	0.1579	^12^
Switzerland°	CH	2013	100	98.3	0.0	1.7	0.3	0.0	11.0	87.0	0.1137	^12^
Turkey	TR	2013	0	87.0	13.0	0.0	22.9	20.9	24.8	18.4	0.1162	^12^
Ukraine^(c)^	UA	2015	NA	52.7	47.3	0.0	20.3	16.2	16.2	0.0	0.0433	^37^
United Kingdom°	GB	2014	NA	100.0	0.0	0.0	0.0	0.0	43.0	57.0	0.3285	^12^

^Caution should be exerted for these country statistics as different international institutes report contrasting figures.

*Under United Nations Security Council Resolution 1244/99.

^(a)^Connected rate was derived from JMP (2018); attribution to treatment levels was based on national statistics.

^(b)^World Bank reports indicate connected rate and percentage of treated waste but not division between treatment levels. In this case half of the rate was attributed to primary and half to secondary level.

^(c)^Treated rate from national statistics; half of the rate was attributed to primary and half to secondary level

^$^IT data for Collected in sewer from year 2009.

^#^PT data for IAS of year 2005.

^°^These countries reported at least 97.5% of domestic waste as treated through IAs or WWTPS and were selected to establish the mean European PE/inhabitant rate.

^+^Data source: Bouraoui *et al*.^1^.

**Online-only Table 2:** Fields of CCM2 catchment layer provided in the dataset.

Field Name	Type	Description	Unit of measure
WSO1_ID	Int	CCM 2.1 elementary catchment identifier	
PE_method	text	Method used to estimate domestic waste (PE_IAS to PE_3P items): REP = UWWTD database; POP = population density and statistics	REP/POP
Pop_SD	Int	Domestic waste Population in scattered dwellings (in this case 1 inhabitant = 1 PE)	PE/y
PE_0		Domestic waste in Population Equivalent that is connected to sewerage but not treated; it is equivalent to the sum of PE_RES_0 and PE_AGGL_0	
PE_RES_0		Domestic waste Population Equivalent (1 PE = 1.23 inhabitant) attributed to connected but not treated population estimated to be unreported in the UWWTD database (part of residual population; see methods section)	
PE_Aggl_0		Domestic waste Population Equivalent reported in the UWWTD database agglomeration table as connected but not treated	
PE_IAS		Domestic waste Population Equivalent treated in Independent Appropriate Systems	
PE_1		Domestic waste Population Equivalent treated in primary treatment WWTPs	
PE_2		Domestic waste Population Equivalent treated in secondary treatment WWTPs	
PE_3		Domestic waste Population Equivalent treated in tertiary treatment WWTPs	
PE_3P		Domestic waste Population Equivalent treated in tertiary treatment WWTPs equipped with phosphorus removal technology	
PE_TOT		Total domestic waste Population Equivalent in the area, is the sum of Pop_SD, PE_IAS, PE_0, PE_1, PE_2, PE_3, and PE_3P	
N_PE	Double	Annual nitrogen waste per PE, dependent on human diet and based on FAO protein consume (Data citation 6)	t/y
N_SD	Double	Domestic waste emissions of nitrogen from scattered dwellings. In REP approach region these are part of ‘residual population’	t/y
N_0		Domestic waste emissions of nitrogen from connected not treated population; it is the sum of N_RES_0 + N_Aggl_0	
N_RES_0		Domestic waste emissions of nitrogen from connected not treated population as unreported in the UWWTD database and estimated as part of residual population	
N_Aggl_0		Domestic waste emissions of nitrogen from connected not treated population from reported UWWTD database agglomeration table as connected but not treated waste	
N_IAS		Domestic waste emissions of nitrogen from Independent appropriate systems	
N_1		Domestic waste emissions of nitrogen from primary WWTPs	
N_2		Domestic waste emissions of nitrogen from secondary WWTPs	
N_3		Domestic waste emissions of nitrogen from tertiary WWTPs	
N_3P		Domestic waste emissions of nitrogen from tertiary with Phosphorus removal WWTPs	
P_PE		Annual phosphorus waste per PE, dependent on human diet and based on FAO protein consume (Data citation 6)	
P_SD		Domestic waste emissions of phosphorus from scattered dwellings. In REP approach region these are part of ‘residual population’	
P_0		Domestic waste emissions of phosphorus from connected not treated population; it is the sum of N_RES_0 + N_Aggl_0	
P_RES_0		Domestic waste emissions of phosphorus from connected not treated population as unreported in the UWWTD database and estimated as part of residual population	
P_Aggl_0		Domestic waste emissions of phosphorus from connected not treated population from reported UWWTD database agglomeration table as connected but not treated waste	
P_IAS		Domestic waste emissions of phosphorus from Independent appropriate systems	
P_1		Domestic waste emissions of phosphorus from primary WWTPs	
P_2		Domestic waste emissions of phosphorus from secondary WWTPs	
P_3		Domestic waste emissions of phosphorus from tertiary WWTPs	
P_3P		Domestic waste emissions of phosphorus from tertiary with Phosphorus removal WWTPs	
BOD_PE		Annual 5-days Biochemical Oxygen Demand waste per PE, dependent on human diet and based on FAO protein consume (Data citation 6)	
BOD_SD		Domestic waste emissions of 5-days Biochemical Oxygen Demand from scattered dwellings. In REP approach region these are part of ‘residual population’	
BOD_0		Domestic waste emissions of 5-days Biochemical Oxygen Demand from connected not treated population; it is the sum of N_RES_0 + N_Aggl_0	
BOD_RES_0		Domestic waste emissions of 5-days Biochemical Oxygen Demand from connected not treated population as unreported in the UWWTD database and estimated as part of residual population	
BOD_Aggl_0		Domestic waste emissions of 5-days Biochemical Oxygen Demand from connected not treated population from reported UWWTD database agglomeration table as connected but not treated waste	
BOD_IAS		Domestic waste emissions of 5-days Biochemical Oxygen Demand from Independent appropriate systems	
BOD_1		Domestic waste emissions of 5-days Biochemical Oxygen Demand from primary WWTPs	
BOD_2		Domestic waste emissions of 5-days Biochemical Oxygen Demand from secondary WWTPs	
BOD_3		Domestic waste emissions of 5-days Biochemical Oxygen Demand from tertiary WWTPs	
BOD_3P		Domestic waste emissions of 5-days Biochemical Oxygen Demand from tertiary with Phosphorus removal WWTPs	
NUTS3_ID		Identifier of administrative unit (level 3) to which the catchment is assigned (Data Citation 7 and Data Citation 8)	
